# Genomic signatures and prognosis of advanced stage Chinese pediatric T cell lymphoblastic lymphoma by whole exome sequencing

**DOI:** 10.3389/fped.2023.1224966

**Published:** 2023-08-16

**Authors:** Qinglin Liu, Xiang Yu, Jinquan Wen, Nange Yin, Xin Liao, Pinli Zou, Yuxia Guo, Lin Song, Jianwen Xiao

**Affiliations:** ^1^ First Clinical College of Chongqing Medical University, Chongqing, China; ^2^ Ministry of Education Key Laboratory of Child Development and Disorders, Chongqing, China; ^3^ National Clinical Research Center for Child Health and Disorders, Chongqing, China; ^4^ Chongqing Key Laboratory of Pediatrics, Chongqing, China; ^5^ Department of Pediatric Hematology, Hospital of Xianyang Caihong, Shaanxi, China; ^6^ Department of Pharmacy, Children’s Hospital of Chongqing Medical University, Chongqing, China; ^7^ Department of Hematology, Children’s Hospital of Chongqing Medical University, Chongqing, China; ^8^ China International Science and Technology Cooperation Base of Child Development and Critical Disorders, Chongqing, China

**Keywords:** T cell lymphoblastic lymphoma, children, whole exome sequencing, PI3K-Akt pathway, USP34 gene

## Abstract

**Objective:**

To investigate the genomic signatures and prognosis of advanced-stage T cell lymphoblastic lymphoma (T-LBL) and to examine the relationship between T-LBL and T cell acute lymphoblastic leukemia (T-ALL).

**Methods:**

35 Chinese T-LBL children with stage III or IV disease were recruited for this study. They were treated with combination chemotherapy and whole exome sequencing. The relationship of the clinical features, prognosis and specific gene mutations was researched. Gene chips of T-LBL and T-ALL were downloaded from a database, and differential gene expression was analyzed.

**Results:**

Germline causal gene mutations (CARS or MAP2K2) were detected in 2 patients; 3.06 ± 2.21 somatic causal gene mutations were identified in the 35 patients, and somatic mutations were observed in the NOTCH1, FBXW7, PHF6 and JAK3 genes. NOTCH1 mutations were signiﬁcantly associated with FBXW7 mutations, and the age at diagnosis of patients with NOTCH1-FBXW7 mutations was less than that of patients without such mutations (*P* < 0.05). 32 patients achieved complete remission (CR), and 14 and 18 patients were classified into the intermediate risk (IR) group and high risk (HR) group. During a median follow-up of 44 months, 3 patients relapsed. Three-year prospective event free survival (pEFS) was 82.286%, and no significant differences of pEFS were found for different sexes, ages, or statuses of NOTCH1-FBXW7 mutations, (*P* > 0.05); however, the mean survival time of the IR group was longer than that of the HR group (*P* < 0.05). Differential expression of genes in the T-LBL and/or T-ALL datasets was analyzed using the R package limma, and 1/3 of the differentially expressed genes were found in both the T-ALL and T-LBL datasets. High expression of PI3K-Akt signal pathway genes and the USP34 gene was found in the T-LBL dataset.

**Conclusion:**

Although T-ALL and T-LBL both originate from precursor T-cells and are considered different manifestations of the same disease and the outcome of T-LBL is favorable when using T-ALL-based chemotherapy, there are differences in the gene distribution between T-LBL and T-ALL. It seems that the PI3K-Akt signaling pathway and the USP34 gene play important roles in T-LBL, but medicines targeting the USP34 gene or the PI3K-Akt pathway may be invalid.

## 1. Introduction

Lymphoblastic lymphoma (LBL) is an aggressive non-Hodgkin's lymphoma (NHL) that accounts for 20%–30% of the pediatric NHL population (1). T cell LBL (T-LBL) involves the precursor T-cell immunophenotype and represents 70%–80% of LBL cases in children (1, 2). The prognosis of T-LBL in children has remained poor historically. Acute lymphoblastic leukemia (ALL)-based chemotherapy and allogenic hematopoietic stem cell transplantation have demonstrated effective results, and event-free survival (EFS) exceeds 80%–90% even for advanced stage (stage III and IV) patients (2, 3), but the survival of relapsed and/or refractory cases remains poor (<10%–30%).

Gene mutations, such as *FBXW7*, *NOTCH1*, and *PTEN* mutations, play important roles in T-LBL development and are potential prognostic biomarkers for T-LBL (2, 4). Pathogenetic and molecular biological studies of T-LBL are limited due to the limitations of detection methods and sufﬁcient available materials.

Next-generation sequencing (NGS) technologies have become the method of choice for gene mutation analysis in cancer (4, 5), but NGS data from Chinese pediatric T-LBL patients have not yet been reported. In the current study, whole exome sequencing (WES) by NGS was used to analyze T-LBL patients with the aim of gaining detailed genome-wide insight into the signature and mechanisms of tumorigenesis in pediatric T-LBL. The relationship of prognosis and specific gene mutations in children with T-LBL was also researched. Gene chips were downloaded from a database, and differential gene expression was analyzed.

## 2. Methods

### 2.1. Patients and treatment

Patients with newly diagnosed T-LBL admitted to Children's Hospital of Chongqing Medical University (CHCMU) and Children's Hospital of Xianyang (CHX) between January 2013 and September 2020 were enrolled in the study. Diagnosis of T-LBL was in accordance with the World Health Organization (WHO) criteria of 2008 or 2016 (6, 7), and patients were staged with the revised international pediatric NHL staging system (IPNHLSS) (8). Patients who were ≥18 years at diagnosis, diagnosed with mixed-phenotype LBL or secondary lymphoma, or had human immunodeﬁciency virus infection were excluded; patients who were classified with a local stage (stage I or II) and who had received chemotherapy before hospitalization were also excluded from the study.

Pathologic diagnosis of T-LBL patients was confirmed by lymph node biopsy. Immunohistochemical staining was detected as described in the literature (7); fluorescence *in situ* hybridization (FISH) of MYC, MLL and SIL-TAL was also performed (9, 10). Blast cells from bone marrow (BM) samples <25% was defined as the cutoff value between T-LBL (stage IV) and T-ALL (1, 6).

Patients were treated with the modified LBL-1995 Berlin-Frankfurt-Münster protocol (BFM-LBL-95) (4, 11). An intrathecal injection was administered as the protocol required, and cranial radiotherapy was carried out for patients with central nervous system (CNS) involvement. Treatment response was evaluated at three time points (TP): TP1 or TP2 (days 15 or 33 of remission induction) and TP3 (prior to consolidation). Minimal disseminated disease (MDD) levels were detected in BM samples using computed tomography (CT) or positron emission tomography computed tomography (PET-CT) screening as described in previous literature reports, and disease status was evaluated as partial remission (PR), complete remission (CR), progressive disease (PD) or refractory disease (RD) (12–14). All patients with advanced stage were regarded as the intermediate risk (IR) group at diagnosis; patients who presented with PD at TP1, PR at TP1 and CR at TP2 were considered as the high risk (HR) group; patients who did not achieve CR at TP3 were considered as having RD.

The details of the risk group classification, course of treatment and drug dosage for the modified BFM-LBL-95 protocol are listed in the Supplementary Material. Clinical data, laboratory findings and prognosis data of enrolled patients were collected and analyzed retrospectively.

### 2.2. DNA isolation and sequencing

Tumor DNA samples of T-LBL patients were obtained at diagnosis from formalin-fixed specimens; germline samples were collected from the oral mucosa of patients and their parents' peripheral blood (PB). Genomic DNA was extracted using a QIAmp DNA Minikit (QIAGEN, China). Genomic DNA was enriched, and sequencing was carried out (Agilent SureSelect Human All Exon V6). PCR products of the whole exome were sequenced (Illumina HiSeq PE 150 bp).

The original WES sequencing data were read using Illumina Pipeline software (version 1.3.4), and data were obtained from databases (dbSNP, 1,000 Genomes Project, ClinVar, ESP6500, ExAc, Ensembl, HGMD, UCSC, etc.). Mutated genotypes were determined using GATK, LRT, Mutation Taster and SamTools software.

The identified variants were divided into the following four categories according to previous literature reports (15) and software analysis: (1) pathogenic genotypes that were confirmed by literature reports; (2) likely pathogenic genotypes that were reported in literature reports and/or affected proteins by function prediction; (3) indefinite variants and (4) single nucleotide polymorphisms (SNPs) or single nucleotide variants (SNPs). Pathogenic genotypes and likely pathogenic genotypes were recorded as causal gene mutations, and causal gene mutations of tumor samples were confirmed by Sanger sequencing. Germline samples were cross-checked and detected by Sanger sequencing, and causal somatic or germline gene mutations were identified.

### 2.3. Identification of DEGs between the T-LBL and T-ALL/ALL datasets

The gene chip dataset GSE29986 was downloaded from the GEO database (https://www.ncbi.nlm.nih.gov/geo/). These datasets included 20 T-LBL samples, 10 T-ALL samples and 6 ALL samples. Differential expression analysis was performed using the R package limma. First, we performed differential analysis of the T-LBL and T-ALL datasets, and we obtained upregulated and downregulated genes in the datasets (adj.*P*. Val <0.05 and |logFC|≥1). Next, we performed differential analysis of the T-LBL and ALL datasets, and we identified upregulated and downregulated genes in these two datasets using the same filtering threshold. Volcano plot showing differentially expressed genes (DEGs) was generated using R language ggplot2. We determined the overlapping genes from the previous two rounds of differential analysis. The overlapping upregulated and downregulated genes related to T-LBL were selected for downstream analysis.

### 2.4. WGCNA of the 36 samples

We used R package weighted gene coexpression network analysis (WGCNA) to construct coexpression modules. Thirty-six samples were used to calculate *Pearson's* correlation coefficients. A power of 6 was selected. An unsigned hybrid coexpression network was then generated using the standard settings. We selected 5,000 genes to construct a topological heatmap. We performed *Pearson* correlation analysis between the module eigengenes and the trait data to identify module-trait relationships. Finally, we selected turquoise module (related to T-LBL) genes to construct a gene regulatory network and performed Gene Ontology (GO) enrichment analysis.

### 2.5. GO and KEGG pathway enrichment analyses

The R package clusterProfiler was used to analyze the GO enrichment of the upregulated gene and downregulated genes related to T-LBL. Biological process (BP) analysis, cellular component (CC) analysis, molecular function (MF) analysis, and Kyoto Encyclopedia of Genes and Genomes (KEGG) pathway enrichment analysis of the selected genes were carried out. After performing WGCNA, the hub genes related to T-LBL were selected as input genes for BiNGO. We used Cytoscape software to visualize the results.

### 2.6. Validation of hub gene expression with TCGA and CCLE databases

To verify the hub genes (USP34, C3 and MGP) identified in WGCNA, we explored the expression level of the hub genes in TCGA (tumor datasets). We checked the expression level of a hub gene (USP34) in different cancers in the CCLE database (https://portals.broadinstitute.org/ccle/home).

### 2.7. Statistical analysis

Events were defined as each of the following situations (2, 4): RD at TP3, relapsed disease, death or diagnosis of a secondary malignancy, or loss to follow-up. With follow-up to December 2020, data on the clinical features, laboratory findings, WES sequencing, treatment responses, CR rate, treatment-related mortality (TRM) and prospective event-free survival (pEFS) of the patients were collected and analyzed.

EFS was calculated from the date of diagnosis to the last follow-up, loss of follow-up or first event. *SPSS 19.0* (IBM Corp., Armonk, NY) software was applied for statistical analysis. Survival curves were calculated according to the *Kaplan-Meier* test. Proportional differences between patient groups were analyzed by *Pearson* chi-squared (*χ*^2^) tests or *Fisher's* exact tests. A *P* value <0.05 was regarded as a significant difference.

## 3. Results

### 3.1. Clinical and laboratory characteristics

Sixty patients with newly diagnosed T-LBL were admitted in the study period; 2 patients were classified as stage I or stage II, and chemotherapy was refused by 4 patients because of family choice. Twelve patients received chemotherapy, but WES sequencing failed because of insufﬁcient available tumor samples. WES sequencing was refused by 7 families. Thirty-five patients received chemotherapy, and WES sequencing was also performed.

The 35 enrolled patients included 25 patients with stage III disease and 10 patients with stage IV disease; 26 males and 9 females were included; family members with gastric cancer were found for 2 (5.71%) patients. Age at diagnosis was 15–168 m (median value: 86 m; average value: 92.83 ± 45.09 m); serum level of lactate dehydrogenase (LDH) was 180–1,947.1 ^U^/L (median value: 339.6 ^U^/L, average value: 522.14 ± 462.40 ^U^/L, normal value <220 ^U^/L), ≥twofold normal level of LDH was detectable in 16 (45.71%) patients; mediastinal, BM or testicular involvement was found in 32 (91.43%), 10 (28.57%) or 1 (2.86%) patients, respectively; and CNS involvement was undetectable in the cohort (Table 1).

**Table 1:** Clinical features of the 35 enrolled patients.

Clinical feathers	*n*=	%
Age (mean ± SD)	92.83 ± 45.09	
<120 m	24	68.57%
≥120 m	11	31.43%
Sex		
Male	26	74.29%
Female	9	25.71%
LDH (mean ± SD, ^U^/L)	522.14 ± 462.40	
<2N	19	54.29%
≥2N	16	45.71%
BM involvement	10	28.57%
Mediastinum involvement	32	91.43%
Testicular involvement	1	2.86%
Stage		
Stage III	25	71.43%
Stage IV	10	28.57%

BM, bone marrow; LDH, lactate dehydrogenase; N, normal value.

### 3.2. Results of WES sequencing

**Figure 1:**
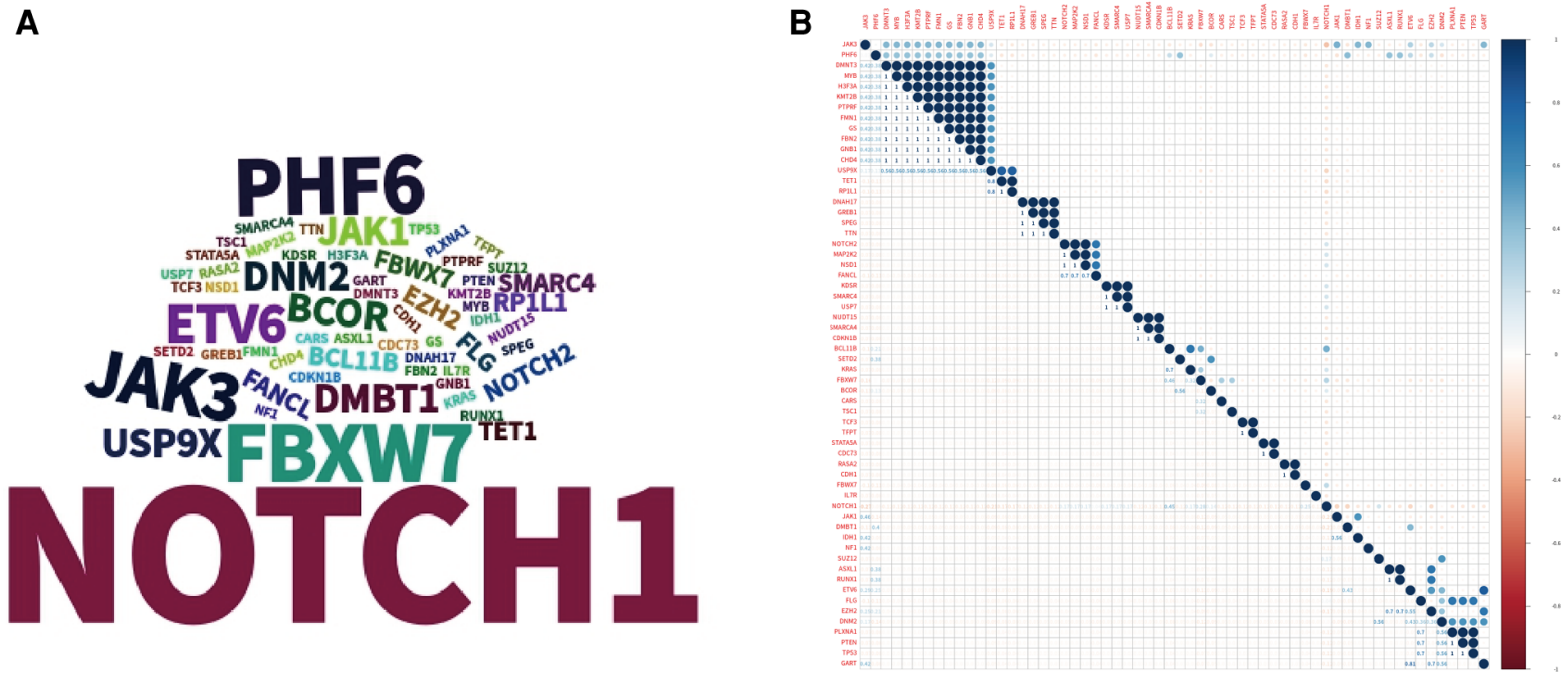
Landscape of gene mutations in the cohort. (**A**) Tagcloud show candidate genes identified by WES analysis word size according to the frequency of the gene. (**B**) Heatmap shows the correlations of mutated genes identified by WES.

WES sequencing of the 35 patients was performed, and germline causal gene mutations (*CARS* or *MAP2K2*) were detected in 2 patients without a family history of cancer; 1–13 (average 3.06 ± 2.21; median 2) somatic causal gene mutations were identified in the 35 patients, and the somatic mutations were observed in the *NOTCH1*, *FBXW7*, *PHF6* and *JAK3* genes (Figure 1A). The relationship among these gene mutations was demonstrated by corplot (Figure 1B), and *NOTCH1* mutations were signiﬁcantly associated with *FBXW7* mutations (6/35, 17.14%). The associations between *NOTCH1-FBXW7* mutational status and clinical characteristics are listed in Table 2, which revealed that the age at diagnosis of patients with *NOTCH1-FBXW7* mutations was less than that of patients without such mutations (*P *< 0.05), whereas significant differences were not found for sex distribution, LDH level or disease staging distribution (*P *> 0.05). These results were similar to those for pediatric T-ALL in previous literature reports (16, 17).

**Table 2:** Relationship between clinical characteristics and somatic mutations.

Clinical characteristics	*NOTCH1-FBXW7* (*n* = 14)	Non *NOTCH1-FBXW7* (*n* = 21)	*P*=
Age (mean ± SD)	77.50 ± 12.13	103.05 ± 9.37	0.0345
Sex (M/F)	11/3	15/6	0.7115
LDH (^U^/L)	657.0 ± 157.1	432.2 ± 224.8	0.1620
Stage (III/IV)	12/2	13/8	0.2516

### 3.3. Treatment evaluation and prognosis

**Figure 2:**
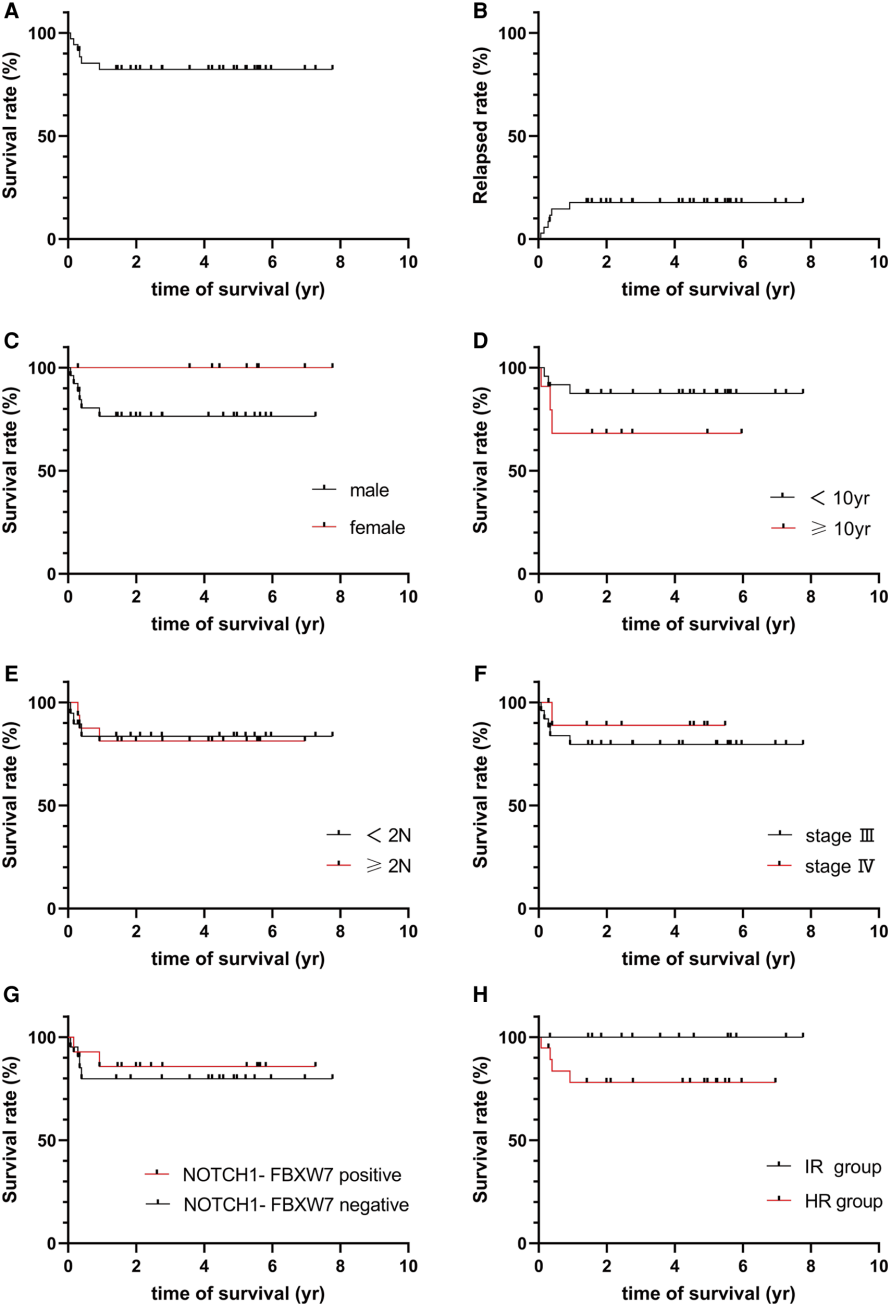
(**A**) Survival of total group. (**B**) Relapsed rate of total group. (**C**) Survival for gender groups. (**D**) Survival for age groups. (**E**) Survival for LDH level. (**F**) Survival for different stage. (**G**) Survival for NOTCH-FBXW7. (**H**) Survival for risk group.

Treatment effects were evaluated at different TP levels as the protocol required. Twelve, 21 or 2 patients were classified as CR, PR or PD at TP1 and TP2, respectively; at TP3, 1 PR patient died of sepsis, 32 achieved CR, and 2 patients were classified as PD, and the CR rate was 91.43% after they finished the course of remission induction. The 2 PD patients quit the study, and 14 and 18 patients were classified into the intermediate risk (IR) group or high risk (HR) group; chemotherapy was continued with the different risk group strategy (Table 3). With follow-up to December 2020, 3 patients relapsed. Among the 35 patients in the cohort, TRM was 2.86%, the relapse rate was 8.57%, and 3-year pEFS was 82.286% (95% CI 64.71–91.64%, Figures 2A,B). pEFS was compared among different sexes, ages, LDH levels and disease stages, but no significant differences were found (*P* > 0.05, Figures 2C–E).

**Table 3:** Association of clinical features and risk groups with prognosis.

Clinical characteristics		Mean survival time (m)	*P*=
Sex	Male	66.581 ± 7.294	0.1375
	Female	57.7778 ± 8.68552	
Age	<10 year	81.502 ± 6.202	0.2246
	≥10 year	49.511 ± 10.357	
LDH (^U^/L)	<2N	77.739 ± 8.901	>0.999
	≥2N	68.615 ± 7.989	
Stage (III/IV)	Stage III	74.328 ± 7.459	0.4449
	Stage IV	57.438 ± 7.074	
Risk group	IR group	66.135 ± 7.884	0.0756
	HR group	42.0714 ± 8.45964	

The 35 patients were classified into 2 groups (group A: *NOTCH1-FBXW7* mutation; group B: without the *NOTCH1-FBXW*7 mutation), and the treatment responses and outcomes were also calculated with different mutational statuses. There was no significant difference in the CR rate between the two groups at TP1, TP2 or TP3 (*P *> 0.05, Table 4). One patient in each group remained PD at TP2; the remaining patients were treated as different risk groups, and a significant difference was not found between different mutational statuses or risk groups (*P *> 0.05, Table 4, Figures 2F–H), but the mean survival time of the IR group was longer than that of the HR group (66.14 ± 7.88 vs. 42.07 ± 8.46 m, *P *< 0.05). Literature reports have shown that the *NOTCH1-FBXW7* mutation is related to favorable outcomes (18–20), but our study showed that the diversity of responses might be overcome by intensive chemotherapy; however, a larger sample and multiple centers are needed to verify this hypothesis.

**Table 4:** Association of NOTCH1/FBXW7 mutational status with prognosis.

	NOTCH1-FBXW7	Non NOTCH1-FBXW7	*P*=
TP1 (CR:PR + PD)	6/8	5/16	0.4913
TP2 (CR:PR + PD)	5/9	5/16	0.4913
TP3 (CR:PR + PD)	13^a^/1	20/1	>0.9999
Risk group (IR/HR)^b^	6/6	8/12	0.7178
Mean survival time (m)	74.513 ± 8.143	74.596 ± 8.259	0.6374

^a^ 1 patient died of sepsis.

^b^ 2PD patients quit.

### 3.4. Identification of DEGs between the T-LBL and T-ALL/ALL datasets

**Figure 3:**
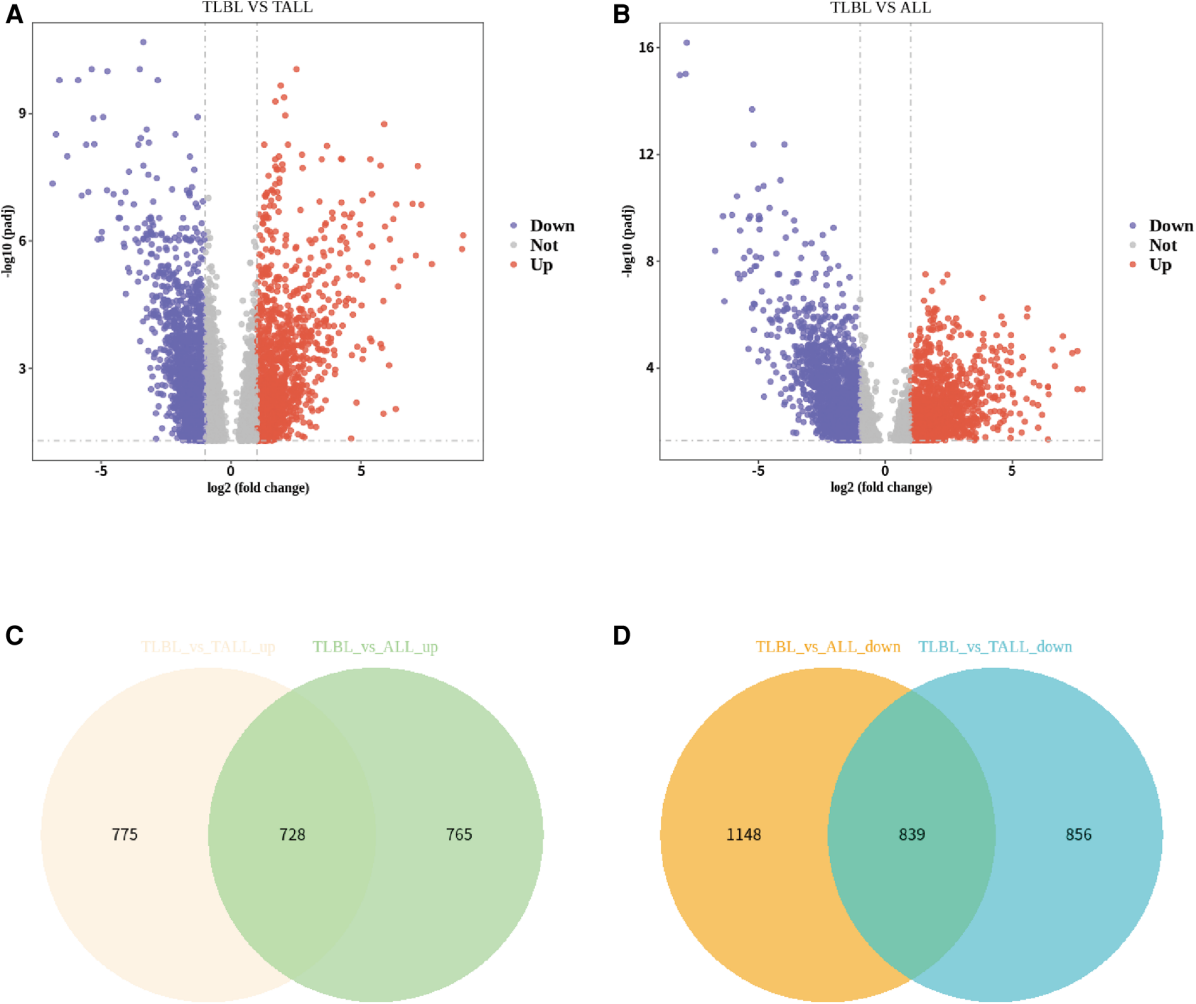
Differential expression analysis of T-LBL versus T-ALL/ALL. (**A**) Volcano plot showed differential expression analysis for TLBL and TALL microarray data. (**B**) Volcano plot showed differential expression analysis for TLBL and ALL microarray data. (**C**) Venn plot showed the overlapped up-regulated genes from differential expression analysis (TLBL versus TALL/ALL). (**D**) Venn plot showed the overlapped down-regulated genes from differential expression analysis (TLBL versus TALL/ALL).

To further understand the pathophysiolopoiesis, differences and similarities of T-LBL and T-ALL, differential expression analysis was performed, and the results were visualized. A volcano plot was generated to show the results of the differential expression analysis; a Venn plot was generated to show the overlapping upregulated and downregulated genes from the differential expression analysis (T-LBL versus T-ALL/ALL, Figures 3A,B). Although T-ALL and T-LBL are considered different manifestations of the same disease and T-LBL is regarded as a nonleukemic phase of T-LBL, it seemed that the gene expression results were not the same between the T-LBL and T-ALL datasets and according to a literature review (21, 22); 1/3 of the differentially expressed genes were found in both the T-ALL and T-LBL datasets.

**Figure 4:**
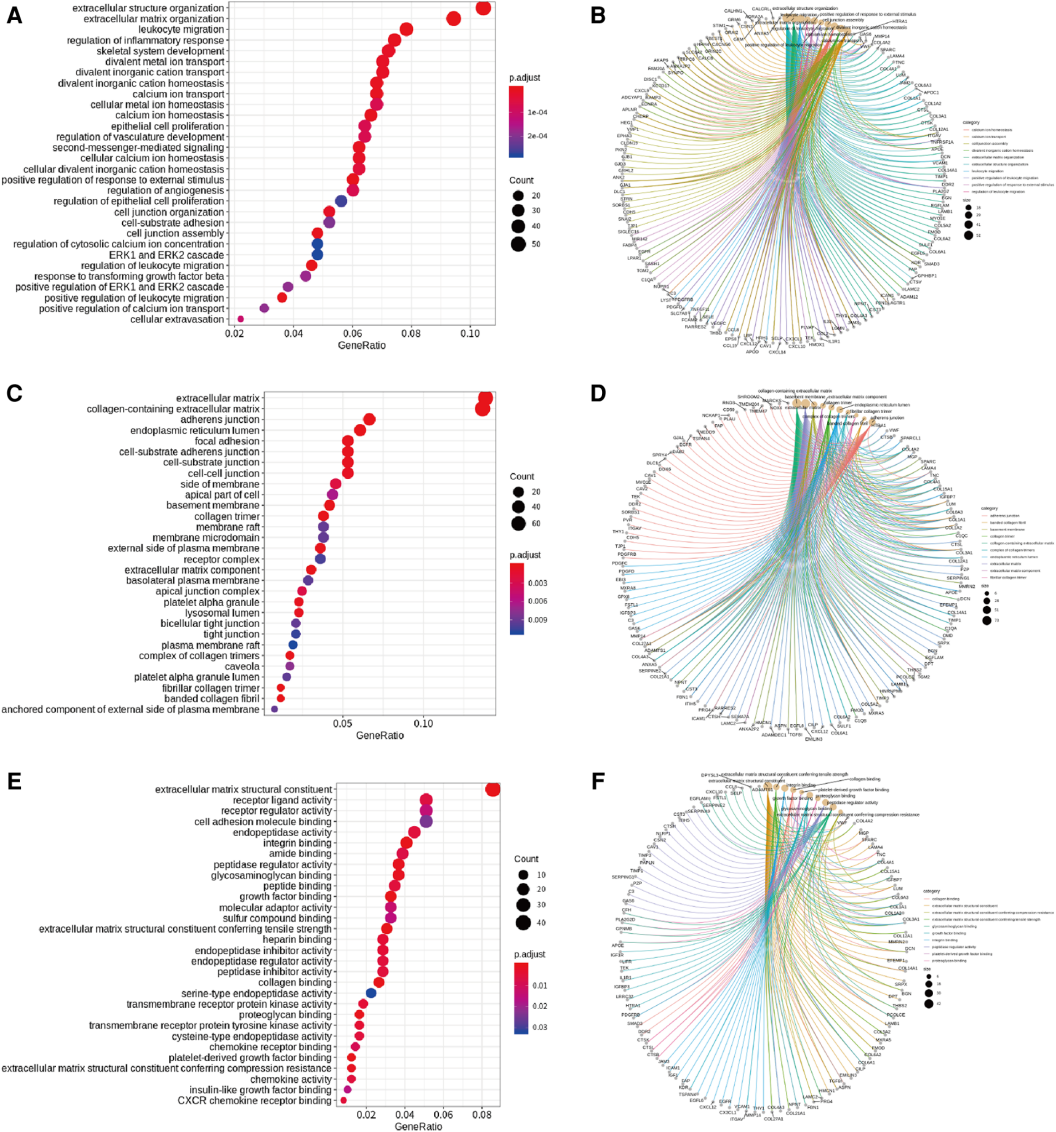
GO and KEGG pathway analyses. (**A, B**) Enriched GO terms (BP) in overlapped TLBL up-regulated genes and the interactions between the gene sets and GO terms. (**C, D**) Enriched GO terms (CC) in overlapped TLBL up-regulated genes and the interactions between the gene sets and GO terms. (**E, F**) Enriched GO terms (MF) in overlapped TLBL up-regulated genes and the interactions between the gene sets and GO terms. (**G, H**) Enriched KEGG pathway in overlapped TLBL up-regulated genes and interaction network between these pathways. (**I, J**) Enriched GO terms (BP) in overlapped TLBL down-regulated genes and the interactions between the gene sets and GO terms. (**K, L**) Enriched GO terms (CC) in overlapped TLBL down-regulated genes and the interactions between the gene sets and GO terms.

Significance was determined as a *P* value of <0.05, and 728 overlapping upregulated genes and 839 overlapping downregulated genes were obtained (T-LBL vs. T-ALL/ALL, Figures 3C,D). The overlapping upregulated genes or downregulated genes related to T-LBL were assessed by GO and KEGG pathway enrichment analyses (BP, CC and MF), and the interactions between the gene sets and GO terms were analyzed and visualized; it appeared that the PI3K-Akt signal pathway, focal adhesion and ECM-receptor interaction play roles in the pathophysiology of T-LBL (Figure 4). However, further studies are required to confirm this result.

**Figure:**
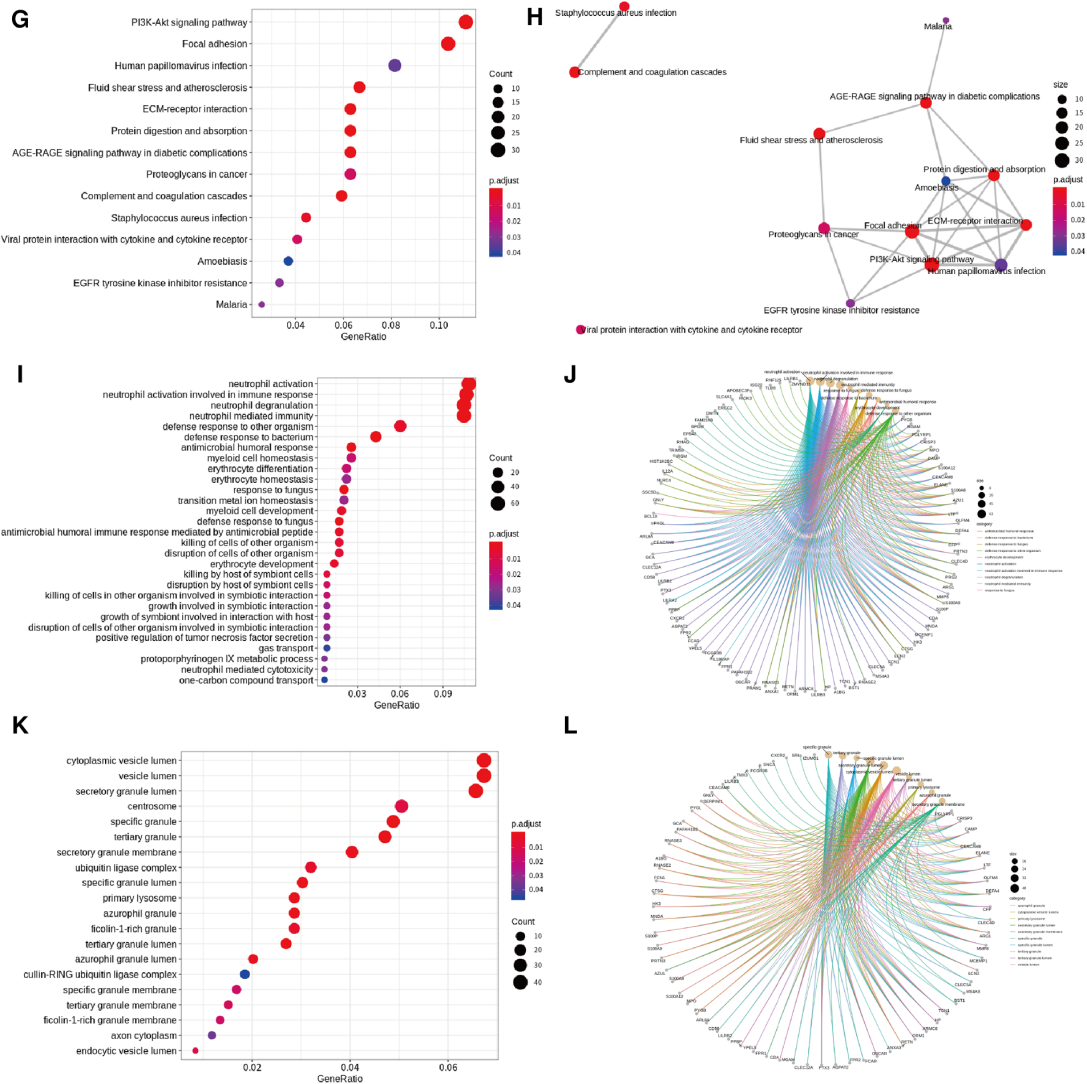


**Figure 5:**
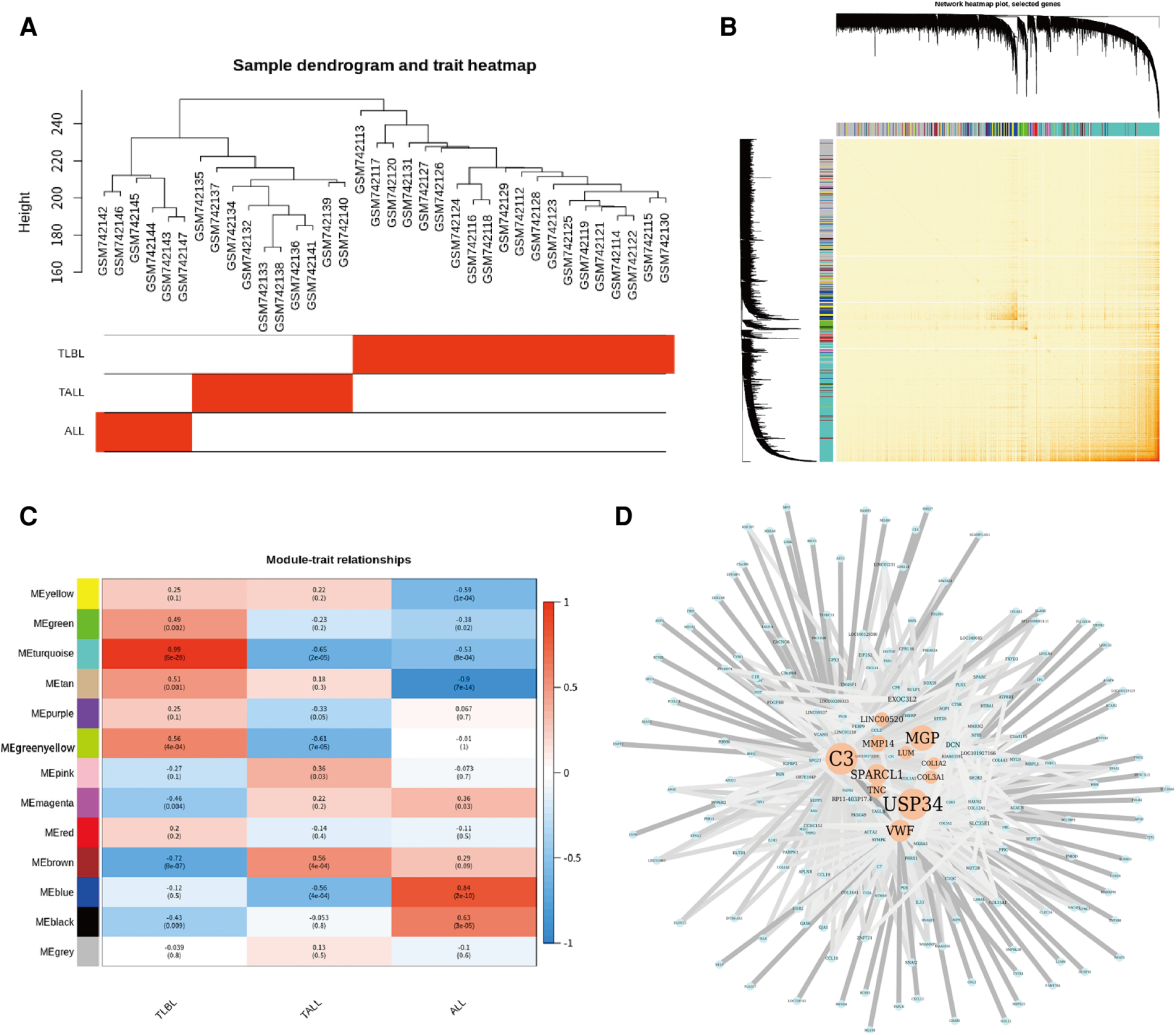
Coexpressed genes in different modules in the top 5000 genes. (**A**) Sample hierarchical clustering plot and trait heatmap from GSE29986 datasets. (**B**) Heatmap showed the Topological Overlap Matrix (TOM) of co-expressed genes in different modules using the top 5000 genes. (**C**) Module and trait relationships. The darker the module color, the more significant their relationship. The turquoise module is most related to TLBL. (**D**) The co-expression network of the significant genes in the turquoise Module (related to TLBL) the color depth of the edge refers to the weight, node size refers to Degree.

**Figure 6:**
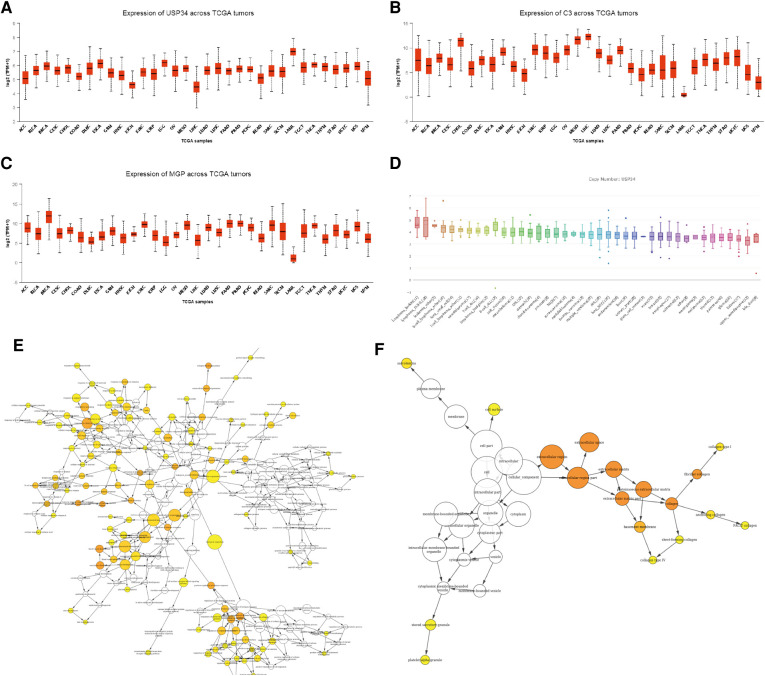
mRNA expression levels of three key genes. (**A-C**) The mRNA expression level of three key genes (USP34, C3 and MGP) based on TCGA tumors data. (**D**) usp34 were high expressed in cancer cell lines from CCLE analysis. (**E**) The gene ontology enrichment (BP) of the significant genes in the turquoise module (related to TLBL). The color depth of the nodes refers to the corrected p values of the ontologies, node size refers to enriched gene numbers. (**F**) The gene ontology enrichment (CC) of the significant genes in the turquoise module (related to TLBL). The color depth of the nodes refers to the corrected p values of the ontologies, node size refers to enriched gene numbers.

Sample hierarchical clustering plots and trait heatmaps from the GSE29986 dataset were generated, and the topological overlap matrix (TOM) of coexpressed genes in different modules of the top 5,000 genes is shown as a heatmap (Figure 5). Module and trait relationships were determined, and the turquoise module was the most related module to TLBL; the coexpression network of significant genes in the turquoise module (related to T-LBL) was generated (Figure 5). The mRNA expression levels of three key genes (*USP34*, *C3* and *MGP*) based on TCGA tumor data were also determined, and the *USP34* gene was highly expressed in the cancer cell lines included in the T-LBL and T-ALL datasets according to CCLE analysis (Figure 6).

Ubiquitination is a common posttranslational modification of proteins and is involved in many physiological activities, such as cell division and differentiation, growth and development, transcriptional regulation, injury stress, and immune response (23). Deubiquitination enzymes have diverse structures and functions; they can remove ubiquitination modifications, affect protein function and regulate physiological activities. The ubiquitin-specific peptidase (USP) superfamily is one of the most widely known deubiquitinase families with the most diverse structures, and the *USP34* gene encodes a member of the USP family that can remove ubiquitin molecules from large protein molecules and eliminate the biological functions of ubiquitinated proteins (24, 25). This USP plays a key regulatory role in the process of DNA damage repair and tumor occurrence and development (24–27).

## 4. Discussion

T-LBL is an aggressive form of non-Hodgkin's lymphoma derived from precursor T cells and makes up approximately 80% of the LBL population. T-ALL is regarded as the leukemic phase of T-LBL (1, 2). Several studies have demonstrated that leukemia-based therapy (e.g., the BFM protocol series) and allo-HSCT are effective and lead to a good prognosis for T-LBL patients, and EFS exceeds 80%–90% for advanced stage patients in developed or developing countries (2, 3, 20, 28). Traditional prognostic factors have been challenging because age, sex, and multiple organ involvement have not been found to be prognostically signiﬁcant by the literature (2, 3) or our study. Germline mutations (*CARS* or *MAP2K2* mutation) were detected in 2 patients without cancer history and the relationship between tumor development and hereditary should be considered, but data were limited, further data were needed to investigate it.

Multiple gene mutations play different roles in the development, progression and carcinogenesis of T-LBL and T-ALL. Our data and the literature suggest that the common somatic mutations are *NOTCH1*, *FBXW7* and *PHF6* mutations and that *NOTCH1* mutations are signiﬁcantly associated with *FBXW7* mutations (5, 16, 20, 29). The associations between *NOTCH1-FBXW7* mutational status and clinical characteristics are listed in Table 2, which revealed that the age at diagnosis in patients with *NOTCH1-FBXW7* mutations was less than that in patients without such mutations (*P *< 0.05), whereas significant differences were not found for sex distribution, LDH level or disease stage distribution (*P *> 0.05). The results were similar to those for pediatric T-ALL in previous literature reports (16, 17).

T-LBL and T-ALL both descend from precursor T-cells, and T-ALL is regarded as the leukemic phase of T-LBL. The outcomes of T-LBL are improved by ALL-based chemotherapy, but EFS of T-LBL and T-ALL in our study were different. Three-year pEFS exceeded 80% for advanced-stage T-LBL in our study, whereas 3-year pEFS was 54.1 ± 11.2% in the pediatric T-ALL population in our department (30). This difference was partly due to the different chemotherapy regimens used for the two patient groups, but the different gene distributions of T-ALL and T-LBL also played important roles in the prognosis in our opinion. Data were collected from T-LBL and T-ALL datasets and analyzed. Only one-third of upregulated genes or downregulated genes overlapped in the two datasets, while the other two-thirds did not overlap (Figure 3), revealing that T-ALL and T-LBL are similar but not the same disease. The different gene distributions of T-ALL and T-LBL may partially due to the sample sources in which DNA samples of T-LBL were acquired from formalin-fixed specimens; in In future investigations, DNA samples obtained from fresh biopsy samples were needed to investigate it; in the other hand, different gene distributions of T-ALL and T-LBL might also existed and these differences could be associated with the carcinogenesis and development of the disease. Therefore, treatment strategies need to be adjusted, and further data and research are needed to confirm this hypothesis.

Although *FBXW7*, *NOTCH1* and *PHF6* mutations were the most common somatic mutations in T-LBL patients, other gene mutations and related pathways were also detectable. The T-LBL database was evaluated by GO and KEGG pathway enrichment analyses, and it appeared that the *PI3K-Akt* signaling pathway and *USP34* gene played important roles in T-LBL. Tumor development, proliferation and metastasis are regulated by abnormal cellular signaling pathways, and genes in the phosphoinositide 3-kinase (*PI3K*)/*Akt* pathway are the most frequently altered in human cancers. Aberrant activation of this pathway is associated with cellular transformation, tumorigenesis, cancer progression, and drug resistance (31, 32). The ubiquitin-specific protease 34 (*USP34*) gene and its protein are closely related to development and progression of human cancers. Research has shown that *USP34* overexpression can be detected in diffuse large B cell lymphoma instead of follicular lymphoma, but a significant association has not been identified between the *USP34* protein level and patient survival (33, 34). Pancreatic cancer cell proliferation and migration are promoted by *USP34* overexpression via upregulation of the *Akt* protein, and pancreatic cancer cell apoptosis induced by an *Akt* inhibitor is reversed by *USP34* overexpression (35). Thus, we assume that *USP34* overexpression and the *PI3K-Akt* signaling pathway are associated with T-LBL development and progression, but anticancer therapies targeting the *USP34* gene or the *PI3K-Akt* signaling pathway may be invalid for refractory relapsed T-LBL patients. Further laboratory studies should be performed.

## Data Availability

The datasets presented in this study can be found in online repositories. The names of the repository/repositories and accession number(s) can be found in the article/**Supplementary Material**.
